# Strain-Specific Differences in the Genetic Control of Two Closely Related Mycobacteria

**DOI:** 10.1371/journal.ppat.1001169

**Published:** 2010-10-28

**Authors:** Tania Di Pietrantonio, Carmen Hernandez, Manon Girard, Annie Verville, Marianna Orlova, Adam Belley, Marcel A. Behr, J. Concepción Loredo-Osti, Erwin Schurr

**Affiliations:** 1 McGill Centre for the Study of Host Resistance, Research Institute of the McGill University Health Centre, Montreal, Quebec, Canada; 2 Department of Human Genetics, McGill University, Montreal, Quebec, Canada; 3 Department of Medicine, McGill University, Montreal, Quebec, Canada; 4 Department of Mathematics and Statistics, Memorial University of Newfoundland, St Johns, Newfoundland and Labrador, Canada; Harvard School of Public Health, United States of America

## Abstract

The host response to mycobacterial infection depends on host and pathogen genetic factors. Recent studies in human populations suggest a strain specific genetic control of tuberculosis. To test for mycobacterial-strain specific genetic control of susceptibility to infection under highly controlled experimental conditions, we performed a comparative genetic analysis using the A/J- and C57BL/6J-derived recombinant congenic (RC) mouse panel infected with the Russia and Pasteur strains of *Mycobacterium bovis* Bacille Calmette Guérin (BCG). Bacillary counts in the lung and spleen at weeks 1 and 6 post infection were used as a measure of susceptibility. By performing genome-wide linkage analyses of loci that impact on tissue-specific bacillary burden, we were able to show the importance of correcting for strain background effects in the RC panel. When linkage analysis was adjusted on strain background, we detected a single locus on chromosome 11 that impacted on pulmonary counts of BCG Russia but not Pasteur. The same locus also controlled the splenic counts of BCG Russia but not Pasteur. By contrast, a locus on chromosome 1 which was indistinguishable from *Nramp1* impacted on splenic bacillary counts of both BCG Russia and Pasteur. Additionally, dependent upon BCG strain, tissue and time post infection, we detected 9 distinct loci associated with bacillary counts. Hence, the ensemble of genetic loci impacting on BCG infection revealed a highly dynamic picture of genetic control that reflected both the course of infection and the infecting strain. This high degree of adaptation of host genetics to strain-specific pathogenesis is expected to provide a suitable framework for the selection of specific host-mycobacteria combinations during co-evolution of mycobacteria with humans.

## Author Summary

Susceptibility to mycobacterial infection results from a complex interaction between host and bacterial genetic factors. To examine the effect of host and pathogen genetic variability on the control of mycobacterial infection, we infected a panel of genetically related recombinant congenic (RC) mouse strains with two closely related strains of *Mycobacterium bovis* BCG. Bacterial counts of BCG Russia and BCG Pasteur were determined in the lung and spleen at 1 and 6 weeks following infection and used for genetic analysis. A novel analytical approach was developed to perform genome-wide linkage analyses using the RC strains. Comparative linkage analysis using this model identified a strong genetic effect on chromosome 1 controlling counts of BCG Pasteur at 1 week and of BCG Russia at 1 week and 6 weeks in the spleen. A locus impacting on late BCG Russia counts in the lung and spleen was identified on chromosome 11. Nine additional loci were shown to control bacterial counts in a tissue-, time-, and BCG strain-specific manner. Our findings suggest that the host genetic control of mycobacterial infection is highly dynamic and adapted to the stage of pathogenesis and to the infecting strain. Such a high degree of genetic plasticity in the host-pathogen interplay is expected to favour evolutionary co-adaptation in mycobacterial disease.

## Introduction

The primary cause of tuberculosis is the human pathogenic bacterium *Mycobacterium tuberculosis*. The host cells of *M. tuberculosis* are macrophages and the bacilli have developed numerous adaptations to survive within these powerful immune effector cells. For example, human pathogenic strains of *M. tuberculosis* inactivate microbicidal superoxide via katalase [1], avoid the detrimental effects of iNOS products [2], skew the anti-mycobacterial response in macrophages towards production of anti-inflammatory molecules [3], [4], and favour necrosis over apoptosis [5], [6], [7]. Interestingly, circulating strains of *M. tuberculosis* may differ in their pathogenic potential [8], [9]. Since humans and *M. tuberculosis* have co-evolved over millennia, a question remains if and to what extent *M. tuberculosis* has adapted to genetically distinct hosts. Indeed, two studies conducted in ethnically mixed samples detected a non-random association of *M. tuberculosis* strains with distinct ethnic populations [10], [11]. These observations are supported by the results of several genetic association studies that detected preferential associations between a Toll-like receptor 2 (*TLR2*) polymorphism and tuberculosis meningitis caused by Beijing strains [12], as well as between variants of 5′-lipoxygenase (*ALOX5*) and pulmonary tuberculosis caused by *M. africanum*, but not *M. tuberculosis* [13]. In addition, variants of the immunity-related GTPase M (*IRGM*) were associated with protection from pulmonary tuberculosis due to Euro-American strains of *M. tuberculosis* [14]. Due to the complex interactions of *M. tuberculosis* and humans in exposed populations, it is possible that those results may have been confounded by unrecognized factors. In the absence of independent replication studies, the question of strain specific genetic effects as a consequence of *M. tuberculosis* human co-evolution still awaits testing under carefully controlled conditions.

*M. bovis* Bacille Calmette-Guerin (BCG) strains are phylogenetic descendants of an ancestral BCG stock originally derived from virulent *M. bovis* through *in vitro* propagation [15], [16], [17]. Attenuation of the original BCG stock occurred as a result of deletions in the *M. bovis* genome, specifically the region of difference 1 (RD1) [18], [19]. Loss of RD1 is common across all BCG strains, although additional genetic alterations have been identified for each strain. BCG Russia and BCG Pasteur are among the most phylogenetically distant BCG strains [15]. Genetic events identified in BCG Russia include the deletion of RD Russia (Rv3698) [20], an insertion mutation in the *recA* gene (recA_D140*) [21], and the presence of an IS*6110* element in the promoter region of the *phoP* gene [15], [22]. BCG Pasteur is characterized by the loss of RD2, nRD18, and RD14 [23], [24], [25] as well as a number of single point mutations and duplication events [22], [23], [26], [27]. Phenotypic differences between BCG Pasteur and BCG Russia can therefore be tentatively linked to these known changes in gene content and an unknown number of point mutations. A number of unresolved questions surround the BCG host interplay which is characterized by highly variable host responsiveness. For example, the immunogenicity of the same strain of BCG given to vaccinees of different genetic background can vary tremendously [28], [29] while host responses triggered by different strains of BCG are equally divergent [30]. On a population scale, BCG strains differ in the adverse reactions they trigger [31] and there is evidence that the protective effect of BCG vaccination against tuberculosis meningitis varies among ethnically divergent population groups [32]. Taken together, these data suggest that, similar to tuberculosis susceptibility, host responsiveness may reflect specific host-BCG strain interactions. To test this possibility, we compared the genetic control of closely related strains of BCG in a mouse model of infection.

Recombinant congenic (RC) strains are a set of genetically related inbred strains. In RC strains, discrete chromosomal segments of donor genome (12.5%) are transferred onto a recipient genetic background (87.5%) through a double backcross and corresponding strains are derived by subsequent inbreeding [33]. The AcB/BcA panel used in the present study was derived from a reciprocal double backcross between C57BL/6J and A/J [34], two mouse strains known to differ in their susceptibility to *M. bovis* BCG strain Montreal [35]. Each RC strain is genetically distinct with its own unique genome. The genomes of all RC strains have been mapped extensively and represent frozen replicas of recombinant progenitor genomes with known genomic boundaries of chromosomal segments derived from the two progenitor strains. A major advantage of RC strains over conventional crosses is that any phenotype can be measured repeatedly in genetically identical mice of a RC strain, greatly improving the accuracy of the phenotypic estimates.

In the present study, 35 distinct AcB/BcA strains were infected with a low dose of either BCG Pasteur or BCG Russia. A genetic analysis of the bacillary counts in the spleen and lungs of these strains identified general, as well as tissue- and BCG strain-specific susceptibility loci for BCG infection. These results demonstrated that the host response to mycobacteria reflects a genetically controlled, joint effect of both host and pathogen. Our findings established strain specific effects of the host-mycobacteria interplay in the absence of selective pressure and, therefore, argue in favour of additional host-mycobacterial adaptation during the co-evolution of humans and mycobacteria.

## Materials and Methods

### Mice and ethics statement

A/J and C57BL/6J mice were purchased from the Jackson Laboratory (Bar Harbor, Maine). Thirty-five independent RC strains originally derived from a reciprocal double backcross between the A/J and C57BL/6J progenitors [34] were purchased from Emerillon Therapeutics Inc. (Montreal, Qc.). All mice were housed in the rodent facility of the Montreal General Hospital. Animal use protocols were approved by the Animal Care Committee of McGill University and are in direct accordance with the guidelines outlined by the Canadian Council on Animal Care.

### Bacterial strains

Recombinant BCG Russia (ATCC 35740) and Pasteur (ATCC 35734), were transformed with pGH1, an integrating vector that inserts into the attB site of the mycobacterial genome and that combines a firefly luciferase lux gene cassette, an integrase [int] gene, a MOP promoter, and a hygromycin resistance [Hyg] gene [31]. The pGH1 vector allows for growth on antibiotic-containing media to reduce risk of contamination [36].

### Infection of mice

BCG strains were grown on a rotating platform at 37°C in Middlebrook 7H9 medium (Difco Laboratories, Detroit, Mich.) containing 0.05% Tween 80 (Sigma-Aldrich, St. Louis, Mo.) and 10% albumin-dextrose-catalase (ADC) supplement (Becton Dickinson and Co., Sparks, Md.). At an optical density (OD_600_) of 0.4 to 0.5, bacteria were diluted in phosphate buffered saline (PBS) to 10^5^ colony forming units (CFU)/ml. Mice were injected intravenously with 10^3^ to 10^4^ CFU of BCG in 100 µL of PBS. Inoculum doses were confirmed by plating on Middlebrook 7H10 agar (Difco Laboratories, Detroit, Mich.) supplemented with oleic acid-albumin-dextrose-catalase (OADC) enrichment (Becton Dickinson and Co., Sparks, Md.).

### BCG load in target organs

Infected mice were sacrificed by CO_2_ inhalation after 1 and 6 weeks post-infection. Lungs and spleens were aseptically removed, placed in 0.025% Saponin-PBS, and homogenized mechanically using a Polytron PT 2100 homogenizer (Brinkman Instruments, Westbury, NY). Homogenates were serially diluted tenfold and plated on Middlebrook 7H10 agar supplemented with OADC enrichment and containing hygromycin B (Wisent Inc., St.-Bruno, Qc.). Bacterial enumeration was performed following a six-week incubation at 37°C. For BCG Pasteur infection, a total of 221 and 175 mice were used at the week 1 and 6 time points, respectively. A total of 145 and 189 mice, respectively, were used at 1 and 6 weeks for BCG Russia infection.

### Genotyping

Strains of the AcB/BcA panel were genotyped for 625 microsatellite markers spanning the entire genome with an average distance of 2.6 cM [34]. Based on Build 36.1 of Mouse Genome Informatics (MGI) Mouse Genome Database, six markers with reassigned positions were removed from the current analysis [37].

### Statistical analysis

The first QTL model was the linear model
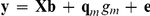



where y represents a vector with the individual total count of bacteria (log_10_CFU); 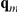 is a vector with each entry being an indicator variable of the genotype BB at the marker position 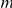 with 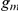 being its associated effect (major gene effect); 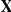 is a matrix of fixed covariates (a constant and gender in our main model) and its corresponding parameter vector 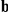; 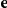 is a vector of independent and identically distributed random variables representing the error term with 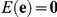 and 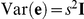. At each marker position 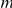, M-estimates of the parameters and a *t*-statistic were computed. The genome-wide corrected *p*-values were obtained by bootstrap under the hypothesis that there is no major gene, i.e., re-sampling under the reduced model
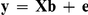



Mean confidence bounds at each marker were defined as twice the standard error around the marker's group mean without considering gender effect in the model.

In order to account for the genetic background, a second linear model of the form
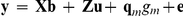



was employed, i.e., our second model was the mixed model resulting from adding a random component, 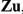 to our original model, where 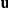 is a random vector associated to the genetic background of each RCS and 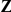 is the design matrix associating the RCS effect to the phenotype **y**. The assumptions for this model component were 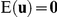 and 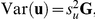 with 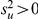 being an unknown constant and 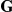 a positive definite-matrix (in fact, a background correlation matrix which is a function of length of the segments identical by descent shared amongst strains) assumed to be known, although a genomic estimate of it was previously obtained. At each marker position 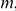 iteratively, estimates of fixed effect parameters and the variance components were obtained under this model and a *t*-statistic of the same form as before was computed. The genome-wide corrected *p*-values were obtained by bootstrap under the hypothesis that there is no major gene, i.e., re-sampling under the reduced model
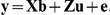



More details of estimation and testing are given in Methods S1. Evidence was considered significant for linkage when single-point regression analysis at the markers was *P*<0.01.

## Results

**Figure 1:**
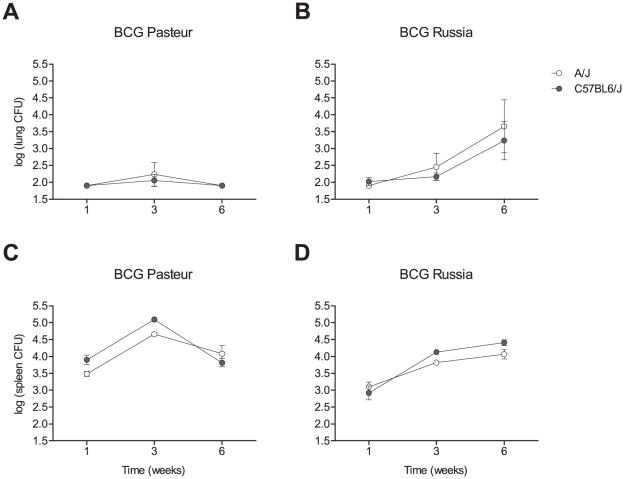
Replication of BCG in the lungs and spleen of A/J and C57BL/6J mice. A/J and C57BL/6J were intravenously infected with a low dose (3–5×10^3^) of either BCG Pasteur (A, C) or BCG Russia (B, D). The number of CFU in the lungs (A, B) and spleen (C, D) was determined at 1, 3, and 6 weeks post-infection. Bacterial counts of BCG Pasteur and BCG Russia were compared in A/J and C57BL/6J mice by two-way ANOVA. Differences in the pulmonary counts of BCG Pasteur and BCG Russia between A/J and C57BL/6J failed to reach significance. However, there was a significant difference in the splenic loads of BCG Pasteur (*P*<0.004) and BCG Russia (*P*<0.0001) between the two strains of mice. These results are representative of at least two experiments. Four to 13 mice were used at each time-point. Data at each time point are the mean log_10_CFU and SD. White, A/J; grey, C57BL/6J.

We determined the bacillary load of BCG strains Pasteur and Russia in the lungs and spleens of C57BL/6J and A/J mice following a low dose (∼3×10^3^ bacilli) intravenous injection of bacilli. Pulmonary counts of BCG Pasteur were below the limit of detectability (80 bacilli/lung) at weeks 1 and 6 post infection but showed a modest peak of approximately 100 bacilli/lung at week 3 (Figure 1). This suggested limited dispersion and growth of BCG Pasteur in the lungs. In addition, there was no detectable difference in the pulmonary load of BCG Pasteur between C57BL6/J and A/J mice. By contrast, we observed an increase of 1–1.5 log CFU in the spleens between weeks 1 and 3 post infection that was followed by a 1 log decrease at week 6. The splenic bacillary burden of BCG Pasteur was substantially higher in C57BL/6J mice at weeks 1 and 3. BCG Russia showed a constant increase of pulmonary CFU from week 1 to week 6. In the spleen, growth of BCG Russia lagged growth of Pasteur and did not show evidence for a peak at 3 weeks post infection, as was observed for Pasteur (Figure 1). Overall, the pattern of tissue CFU for BCG Pasteur strongly resembled the one described for BCG Montreal which has previously been shown to be under *Nramp1* control [35], [38]. The kinetics of lung and spleen bacillary counts of BCG Russia were distinct from the previously described BCG growth patterns.

To investigate the genetic control of *in-vivo* growth of BCG Russia and BCG Pasteur, mice from a panel of 35 AcB/BcA RC strains were intravenously challenged with a low dose (3–5×10^3^ bacilli) of BCG Russia or BCG Pasteur. The number of colony forming units (CFU) in the spleen and lung was used as the phenotype for the genetic analysis. CFU were determined at 1 week and 6 weeks post infection since it is well established that at 3 weeks, the *Nramp1* gene dominates the host response to BCG Montreal [38], making it potentially more difficult to discern additional genetic control elements.

**Figure 2:**
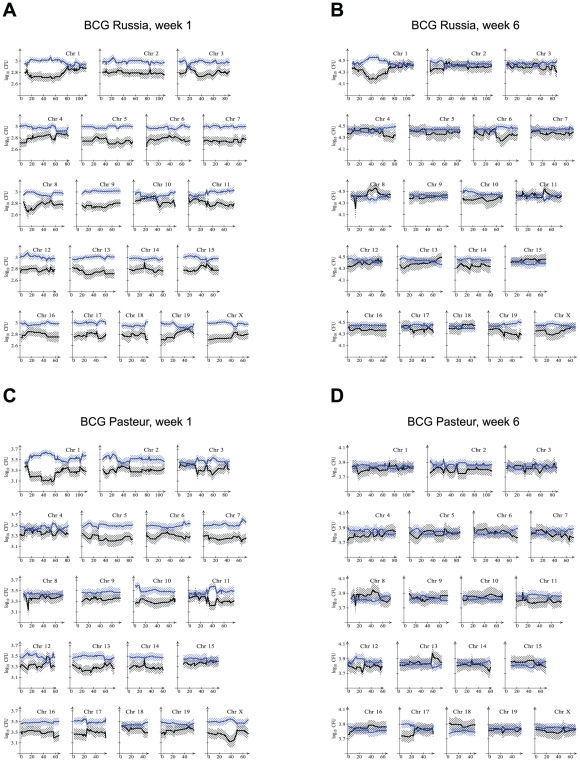
Spleen bacillary counts relative to A/J and C57BL/6J-derived chromosomal segments in RC mice. AcB and BcA mice were infected with BCG Russia (A, B) or BCG Pasteur (C, D) and spleen bacterial counts were determined at 1 week (A, C) and 6 weeks (B, D) post-infection. RC mice were stratified by genotype (AA in black or BB in blue) at each microsatellite marker and the mean log_10_CFU (solid line) as well as twice the standard error confidence bounds (hatched area) were determined for the two groups of mice. Gaps between the mean CFU of the AA and BB genotype are indicative of markers where the two groups differed. Chromosomal positions are given in centimorgans (cM).

**Figure 3:**
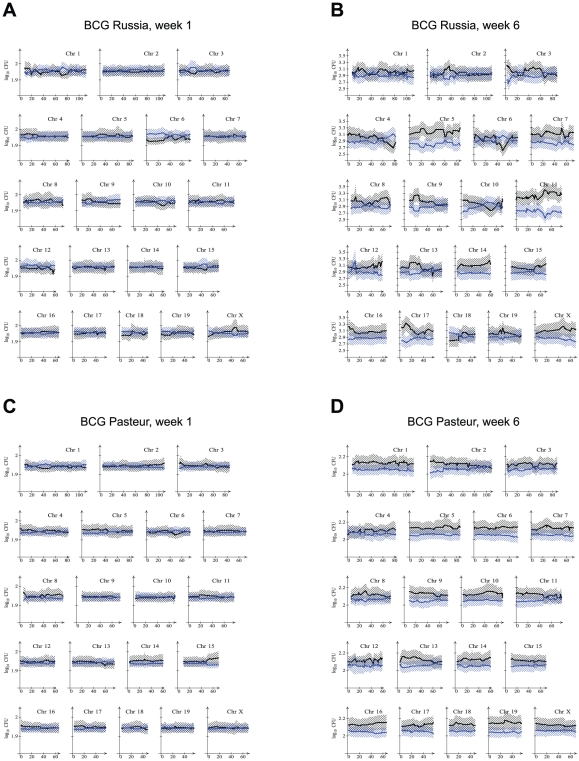
Lung bacillary counts relative to A/J and C57BL/6J-derived chromosomal segments in RC mice. Pulmonary bacterial loads in RC mice intravenously infected with BCG Russia (A, B) or BCG Pasteur (C, D) were determined at 1 week (A, C) and 6 weeks (B, D) post-infection. RC mice were stratified by genotype (AA in black or BB in blue) at each microsatellite marker and the mean log_10_CFU (solid line) and confidence bounds (hatched area) were determined for the two groups of mice. A divergence in the mean CFU of the AA and BB genotype groups represent chromosomal regions where the two groups differed. Chromosomal positions are given in centimorgans (cM).

To best indicate the effect of genotype on CFU, all RCS were stratified according to genotype at each marker, i. e. AA for markers on chromosomal segments derived from A/J or BB for chromosomal segments derived from C57BL/6J. Mice of all RCS with a given genotype were then used to obtain the mean and 95% confidence interval of their pulmonary and splenic CFU. This presentation allowed to graphically depict the effect of both marker genotype and of the general strain background on CFU. Results for the spleen and lung for both BCG strains are presented in Figures 2 and 3. A clear impact of strain background on susceptibility to BCG in the spleen at 1 week post infection was evidenced by the larger bacillary counts in mice of the BB genotype across most chromosomes (Figure 2). The strong strain background effect on splenic CFU was resolved by 6 weeks post infection, particularly for BCG Pasteur where differences in splenic bacillary burden appeared negligible across all markers (Figure 2). By contrast, CFU differences in BCG Russia were observed for several small chromosomal segments possibly suggesting the presence of specific genetic loci (Figure 2). As in the parental strains, pulmonary burdens were at the limit of detectability at week 1 for both Russia and Pasteur, and week 6 for Pasteur. However, at the 6-week endpoint, preferential replication of BCG Russia was observed in mice bearing specific A/J-derived chromosomal segments, particularly at the distal portion of chromosome 11 (Figure 3).

Markers where the mean CFU of the AA and BB genotype groups diverged were indicative of chromosomal regions that potentially harboured a BCG susceptibility locus. To confirm the potential linkage of these chromosomal segments to bacterial burden, a genetic analysis comparing mice of the AA to BB genotype was performed. The initial analysis compared genotype groups without taking into account the genetic background of the strain or the gender of the mouse (incomplete model). As expected, markers significantly linked to bacterial burden corresponded well with chromosomal regions where the two genotypes differed (Figures 2 and 3; Figures S1 to S3). From this analysis, the genetic control of BCG Pasteur and Russia splenic infection appeared to be highly multigenic at the early time point. Employing a very stringent level of significance (*P*<0.0003), quantitative trait loci (QTL) were identified across 8 and 15 different chromosomes for BCG Pasteur and Russia, respectively (Figure S1). At the 6 week endpoint, a locus was identified on chromosome 1 for splenic BCG Russia load whereas genetic effects were not detected for BCG Pasteur load (Figure S2). Pulmonary CFU of BCG Russia was controlled by a locus on chromosome 11 while for BCG Pasteur a locus was identified on chromosome 8 (Figure S3).

**Figure 4:**
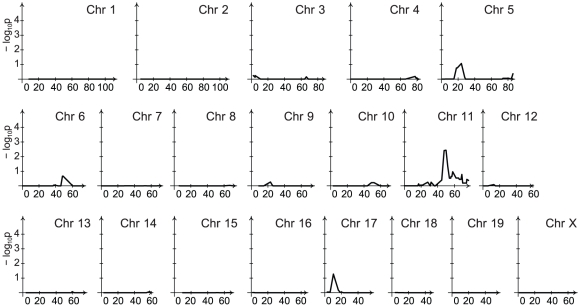
Genetic control of late pulmonary bacillary counts of BCG Russia. Linkage analysis of BCG Russia pulmonary counts at the 6-week time point was performed with background and gender-adjustment. A single locus controlling BCG Russia pulmonary counts was identified on chromosome 11. Chromosomal positions are given in centimorgans (cM).

Visual inspection of CFU across genotypes suggested a strong impact of strain background on bacillary loads. To account for the potential impact of background genes on linkage peaks, we developed a main model that accounted for the genetic background and gender of the mice. The number of loci identified by the main model was reduced relative to the incomplete model, particularly at the 1 week time point (Figures S4 and S5, and Table 1). For lung CFU, the locus on chromosome 11 remained that impacted on bacillary load of BCG Russia at 6 weeks post infection (Figure 4). No genetic effect was detected for pulmonary load of BCG Pasteur which is consistent with the very limited growth of BCG Pasteur in the lungs of all mice (data not shown).

**Table 1:** Summary of significant linkage peaks obtained using the main and conditional models.

Strain	Organ	Day	Main model	Conditional model
BCG Pasteur	Spleen	7	Chrs. 1, 2, 3, 6, 7, 10, 17, X	(Chr. 1)^a^
		42	- b	N/A
	Lung	7	-	N/A
		42	-	N/A
BCG Russia	Spleen	7	Chr. 1	(Chr. 1)
		42	Chrs. 1, 6, 11, 19	Chrs. (1), 11, 13
	Lung	7	-	N/A
		42	Chr. 11	N/A

Chr., chromosome; N/A, not applicable.

a (Chr. 1), exclusion of markers on chromosome 1 to adjust for major genetic effect.

b -, no significant genetic effects detected.

In contrast to the lung, the genetic control of splenic bacillary load remained largely multigenic even after correction for strain background effects. For BCG Russia at 1 week post infection, a single locus on chromosome 1 (36.9 cM–48.8 cM) was found to control splenic load (Figure S4). At 6 weeks post infection, the genetic control of BCG Russia was multigenic (Figure S5). In addition to the chromosome 1 locus (32.8–55.1 cM), loci were detected on chromosome 6 (45.5–46.3 cM), chromosome 11 (47.67 cM) and chromosome 19 (51 cM). Splenic load of BCG Pasteur at 1 week post infection was controlled by loci on chromosome 2 (10–15 and 22.5–26.2 cM), chromosome 7 (63.5–65.6 cM) and the X chromosome (37–40.2 cM). Additional weaker effects were identified on chromosome 3 (33.7 and 58.8 cM), chromosome 6 (63.9 cM), chromosome 10 (3 cM), and chromosome 17 (23.2 cM). A major gene effect detected on chromosome 1 (17–58.5 cM) overlapped the chromosome 1 locus controlling BCG Russia infection (Figure S4, Table 1). Genetic control elements were not detected in response to BCG Pasteur infection at the 6 week time point (data not shown). The inverse complexity of BCG Pasteur (multigenic at 1 week; no genes at week 6) and BCG Russia (a single gene at week 1, multigenic at week 6) reflects differences in the replication pattern of the bacteria: BCG Russia showed a delayed onset of growth that continued at week 6 while BCG Pasteur showed rapid initial growth with a strong decline of CFU at week 6 as compared to week 3.

**Figure 5:**
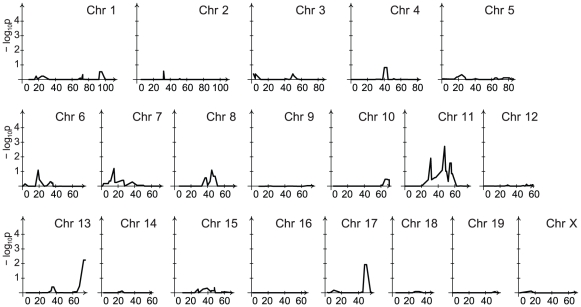
Linkage analysis of late spleen bacillary counts contingent on the chromosome 1 locus. Linkage analysis was performed with an adjustment for the locus on chromosome 1 which had a major effect on the splenic bacillary counts of BCG Russia at the 6-week time point. The loci identified on chromosomes 6 and 19 were conditional upon the chromosome 1 locus and lost their significance when adjusted. The genetic effect detected on chromosome 11 was independent of the chromosome 1 locus and maintained its significance. An additional locus was localized to chromosome 13. Chromosomal positions are given in centimorgans (cM).

The chromosome 1 locus significant for linkage early during BCG Pasteur infection and at the early and late phase of BCG Russia infection was indistinguishable from *Nramp1*. Employing what we termed the “conditional model,” we determined whether the additional linkage peaks were conditional on the *Nramp1* gene. For this the main model was modified to adjust for the effect of *Nramp1* by adding a column with the BB genotype indicator at the *Nramp1* position to the matrix **X**. Chromosomal regions identified at the week 1 time point of both BCG Pasteur and BCG Russia infection were no longer significant for linkage following correction for the chromosome 1 locus (data not shown). Similarly, the genetic effects detected on chromosome 6 and 19 were no longer significant at the 6 week time point of BCG Russia infection. However, the linkage hit detected on chromosome 11 (47.67 cM) retained its significance. By contrast, a secondary peak detected only for splenic CFU immediately proximal to this locus did not reach significance (Figure 5). Finally, an additional locus was localized to chromosome 13 (73–75 cM) (Figure 5).

## Discussion

RC strains are particularly useful to establish pathways of causality in complex read-outs such as immune reactivity and are well suited to track gene-gene interactions [33]. However, RC strains have also proven useful for positional identification of disease susceptibility loci by employing RC strains with extreme phenotypes in subsequent genetic crosses [39], [40], [41]. A third application of RC strains is the genome-wide identification of quantitative trait loci (QTL) in complex diseases. This feature of RC stains is particularly attractive since it allows the measurement of quantitative traits in many genetically identical mice belonging to the same strain which greatly increases the accuracy of trait determination. A genome-wide scan for the presence of QTL can then be conducted among the relatively limited number of RC strains in each panel. This is highly efficient compared to the breeding and genotyping of hundreds of mice in traditional backcross or F2 based genome-wide mapping studies. For example, a recent study used the AcB/BcA RC strain panel to localize a large number of asthma susceptibility loci across the genome [42]. A potential problem that is faced in these speedy genome-wide scans in RC strains is the confounding impact of strain background and of strong susceptibility loci on the overall pattern of QTLs mapped. We have developed a new analytical methodology that overcomes both of these potentially confounding limitations while conducting genome-wide QTL mapping in RC strains. Our results demonstrate the ease of genome-wide scanning in RC strains and the importance of adjusting especially on strain background to achieve reliable QTL identification.

Our ability to detect the *Nramp1* genomic region also served as an internal validation of the analytical approach. Another interesting observation was the loci that could only be detected in connection with *Nramp1*. Once the analysis was adjusted on the *Nramp1* gene, these loci were no longer significant for linkage. The most parsimonious explanation for this effect is that these loci are interacting with *Nramp1*. Why we would detect a large number of genes that interact with *Nramp1* in the genetic control of BCG Pasteur as compared to BCG Russia is not known but may reflect the differences in pathogenesis between the two BCG strains. For BCG Pasteur, putatively interacting genes were detected at 3 weeks post infection while for BCG Russia such interacting loci were observed at the 6 week time point. At 3 weeks, BCG Pasteur shows a sharp peak of splenic bacillary burden while the growth of BCG Russia continues well past 6 weeks before a slow and gradual reduction of splenic burden becomes evident after 12 weeks of infection (data not shown). While the interpretation of our results as *Nramp1* interacting loci appears reasonable, it is important to realize that this conclusion needs further direct experimental validation. However, if correct, the mapping tools presented in this paper would provide a very powerful approach for the identification of interacting loci which is still a major obstacle in complex trait analysis in both human and model animals.

The study of the impact of strain variability of *M tuberculosis* on disease expression is of considerable interest for the implementation of tuberculosis control measures. An increasing body of evidence suggests that different strains/lineages of *M. tuberculosis* display substantial differences in their pathogenic potential [8], [9]. In addition, evidence is emerging that genetic variability among BCG vaccine strains is a potent factor in modulating BCG induced anti-tuberculosis immunity [31]. This mycobacterial strain variability reflects an even greater divergence in host responsiveness to both BCG and *M. tuberculosis* that is largely under host genetic control (reviewed in [43]). These observations raise the question if host and mycobacterial variability are independent of each other. If independent, we would expect hosts to display a spectrum of responsiveness from highly resistant to highly susceptible irrespective of the infecting mycobacterial strain. Similarly, *M. tuberculosis* strains would vary from highly virulent to mildly virulent across all hosts. Alternatively, it is possible that “susceptibility” and “virulence” are not absolute but rather reflect specific combinations of mycobacterial strain and human host. The latter possibility is supported by recent observations of preferential associations of tuberculosis lineages with ethnic groups that may reflect co-adaptation of *M. tuberculosis* and its human host [10]. Moreover, a number of host genetic association studies have reported a preferential association of tuberculosis susceptibility variants with specific *M. tuberculosis* lineages [12], [13], [14]. The results of our study obtained in a highly controlled experimental setting support the hypothesis of host – pathogen specific genetic “fits.” Hence, human susceptibility to tuberculosis may only become tractable by jointly considering host and pathogen genetic backgrounds.

By conducting a genome-wide mapping of loci that impact on the splenic and pulmonary burden following a low dose infection with two strains of BCG, we revealed a divergent pattern of susceptibility loci. An unexpected result was the pronounced dynamic of genetic loci impacting on bacillary counts. This observation demonstrated how different genetic control elements came into play as the BCG infection advanced and further emphasized the intimate interplay between host genetics and pathogenesis. Perhaps less surprising was the large difference in the number of loci involved in the control of splenic vs pulmonary bacillary counts. BCG Pasteur shows little dissemination and growth in the lungs of infected mice and the absence of susceptibility loci was therefore expected. However, BCG Russia reaches bacillary counts in the lungs that are similar to those in the spleen. Yet, only one susceptibility locus on chromosome 11 was detected to impact on pulmonary counts while splenic counts are under more complex control. It is interesting that a locus on chromosome 1 which is indistinguishable from the *Nramp1* gene had by far the strongest impact on bacillary burden in both BCG Pasteur and Russia, but this effect was limited to splenic counts. By contrast, the chromosome 11 locus was detected only for BCG Russia but in both the spleens and lungs. The results therefore indicate that host genetic control is characterized by very strong common control elements that act in a tissue –specific manner, and by somewhat weaker BCG strain specific susceptibility genes that are not tissue specific. Together these data indicate that host genetic control of mycobacterial replication is sensitive to the particular strains but also to differences in disease manifestations (here, lung vs spleen). Interestingly, the strongest genetic effect ever found in human studies was found in an outbreak of tuberculosis in Northern Canada [44]. During this outbreak, all cases had been infected from a single index case, i.e. a single bacterial strain [45]. A fine tuned host genetic response to mycobacteria might explain why it has been difficult to reproducibly detect strong host genetic effects in human tuberculosis. Consequently, future genetic studies of tuberculosis susceptibility might need to be adjusted on the detailed clinical picture and infecting *M. tuberculosi*s strain.

## Supporting Information

Methods S1 Details of estimation, bootstrap and testing.
(0.05 MB PDF)

Figure S1 Linkage analysis of early splenic counts independent of the genetic background. Bacillary counts of BCG Russia and BCG Pasteur in the spleen of RC mice at the week 1 time point were used for QTL analysis. AA and BB genotype groups were analyzed without taking into account the gender or genetic background of the RC mice. Significant evidence for linkage was detected across 15 different chromosomes for BCG Russia (A) and across 8 different chromosomes for BCG Pasteur (B) at the week 1 time point. Chromosomal positions are given in centimorgans (cM).
(1.83 MB TIF)

Figure S2 Linkage analysis of late splenic counts independent of the genetic background. Bacterial numbers of BCG Russia and BCG Pasteur in the spleen of RC mice at the week 6 time point were used for linkage analysis. Significant linkages were detected on chromosomes 1 and 19 for BCG Russia (A) whereas no significant evidence for linkage was detected for BCG Pasteur (B). Chromosomal positions are given in centimorgans (cM).
(1.59 MB TIF)

Figure S3 Linkage analysis of late pulmonary counts independent of the genetic background. QTL analysis was performed using pulmonary counts of BCG Russia and BCG Pasteur at the week 6 time point. Loci controlling pulmonary bacterial numbers were identified on chromosome 11 for BCG Russia (A) and chromosome 8 for BCG Pasteur (B). Chromosomal positions are given in centimorgans (cM).
(1.61 MB TIF)

Figure S4 Genetic control of early spleen bacillary counts of BCG Russia and BCG Pasteur. Linkage analysis of splenic bacterial counts at the week 1 time point was performed with an adjustment for strain genetic background and gender. A single locus on chromosome 1 was identified in response to early BCG Russia infection (A). Loci linked to splenic BCG Pasteur counts were detected on chromosomes 1, 2, 3, 6, 7, 10, 17 and X at the week 1 time point (B). Chromosomal positions are given in centimorgans (cM).
(1.69 MB TIF)

Figure S5 Genetic control of late spleen bacillary counts following infection of the RC strains with BCG Russia. Background- and gender-adjusted QTL analysis was performed using spleen counts of BCG Russia at the 6-week endpoint. A locus on chromosome 1 had a major effect on the bacterial numbers of BCG Russia. Additional loci were detected on chromosomes 6, 11, and 19. Chromosomal positions are given in centimorgans (cM).
(0.73 MB TIF)
