# Supplementary material for: Strain-Specific Differences in the Genetic Control of Two Closely Related Mycobacteria
Source: PLoS Pathog. 2010 Oct 28;6(10):e1001169. doi: 10.1371/journal.ppat.1001169 (PMC2965770; doi:10.1371/journal.ppat.1001169)
Supplement: Methods S1 — Details of estimation, bootstrap and testing. (0.05 MB PDF) [file ppat.1001169.s001.pdf]

## Supplement 1. Methods.

**Estimation.** Without loss of generality, here we describe the estimation for the mixed model

$$\mathbf{y} = \mathbf{Xb} + \mathbf{Zu} + \mathbf{e}$$

where **X**, **Z** are known matrices, **b** is an unknown fixed vector and **u** and **e** are random vectors with null mean and variance $s_u^2\,\mathbf{G}$ and $s^2\mathbf{I}$, respectively. Thus

$$\text{E}(\mathbf{y}) = \mathbf{Xb} \qquad \text{and} \qquad \text{Var}(\mathbf{y}) = s^2\left(\mathbf{ZHZ}' + \mathbf{I}\right) = s^2\,\mathbf{Q}$$

with $\mathbf{H} = k^{-1}\mathbf{G}$ and $k = \frac{s^2}{s_u^2}$. Given **H**, the best linear estimator of **b** and predictor of **u** can be written as

$$\hat{\mathbf{b}} = (\mathbf{W}'\mathbf{W})^{-}\mathbf{W}'\mathbf{v} \quad \text{and} \quad \hat{\mathbf{u}} = \mathbf{HZ}'\mathbf{Q}^{-\frac{1}{2}}(\mathbf{v} - \mathbf{W}\hat{\mathbf{b}}),$$

respectively, where

$$\mathbf{W} = \mathbf{Q}^{-\frac{1}{2}}\mathbf{X} \quad \text{and} \quad \mathbf{v} = \mathbf{Q}^{-\frac{1}{2}}\mathbf{y}.$$

Also

$$\hat{s}^2 = \frac{1}{N - r(\mathbf{W})}(\mathbf{v} - \mathbf{W}\hat{\mathbf{b}})'(\mathbf{v} - \mathbf{W}\hat{\mathbf{b}})$$

$$\hat{s}_u^2 = \frac{1}{r(\mathbf{H})}\left(\hat{\mathbf{u}}'\mathbf{H}^{-1}\hat{\mathbf{u}} + \hat{s}^2 tr(\mathbf{H}^{-1}\mathbf{C})\right)$$

with

$$\mathbf{C} = (\mathbf{Z}'\mathbf{MZ} + \mathbf{H}^{-1})^{-1} \quad \text{and} \quad \mathbf{M} = \mathbf{I} - \mathbf{X}(\mathbf{X}'\mathbf{X})^{-1}\mathbf{X}.$$

The estimation procedure runs as follows: get a starting value for $k$ and iterate through these equations until convergence is reached.

**Bootstrap.** When **b** and $k$ are known, it follows from the model reduced under the null hypothesis that $\text{E}(\mathbf{v}) = \mathbf{Wb}$ and $\text{Var}(\mathbf{v}) = s^2\,\mathbf{I}$, thus the distribution of vector of residuals, $\mathbf{r} = \mathbf{v} - \mathbf{Wb}$, is exchangeable, which means that a resampling procedure based upon the residuals will have good asymptotic properties. This suggests the following semi-parametric bootstrap procedure: i) given $\hat{k}$ and $\hat{\mathbf{b}}$ obtained under the mixed model without a major gene, i.e., under the null hypothesis, compute $\hat{\mathbf{r}} = \mathbf{D}(\mathbf{v} - \mathbf{W}\hat{\mathbf{b}})$ where **D** is a diagonal matrix with each of the non-zero elements given by $(1 - l_{ii})^{-1}$ and $l_{ii}$ being the $i$th the leverage coefficient; ii) with replacement, resample from $\hat{\mathbf{r}}$ to obtain $\mathbf{r}^*$ and construct the pseudo-observation as $\mathbf{v}^* = \mathbf{W}\hat{\mathbf{b}} + \mathbf{r}^*$.

**Testing.** Obtain the genome-wide corrected empirical p-values by the following procedure: i) at each marker position, fit the major gene model

$$\mathbf{v} = \mathbf{W}\mathbf{b} + \mathbf{q}'_m g_m + \mathbf{r}$$

where $\mathbf{q}'_m = \mathbf{Q}^{-\frac{1}{2}}\mathbf{q}_m$ (of course, this transformed model is completely equivalent to the one described in the text) and estimate the model parameters with the mixed model procedure oulined above; ii) compute $\mathbf{t_m}$, the vector whose $m$-th entry is the $t$-statistic, i.e., $t_m = \frac{|\hat{g}_m|}{\hat{s}_{\hat{g}_m}}$; iii) draw a pseudo-observation $\mathbf{v}^*$ by using the previous resampling scheme and fit the major gene model in (i) with $\mathbf{v}$ replaced by $\mathbf{v}^*$ to obtain a pseudo-statistic vector $\mathbf{t}^*_\mathbf{m}$ as well as its associated critical value $t^*_c = \max\ \mathbf{t}^*_\mathbf{m}$; iv) for each $t_m$ in $\mathbf{t_m}$, if $t_m \leq t^*_c$, update the $m$-th rejection count by adding an unit. v) Repeat the steps (iii)-(iv) $B$ times and compute the estimates of p-values by dividing the rejection count vector by $B$.
