# Supplementary figures and images for: Strain-Specific Differences in the Genetic Control of Two Closely Related Mycobacteria
Source: PLoS Pathog. 2010 Oct 28;6(10):e1001169. doi: 10.1371/journal.ppat.1001169 (PMC2965770; doi:10.1371/journal.ppat.1001169)

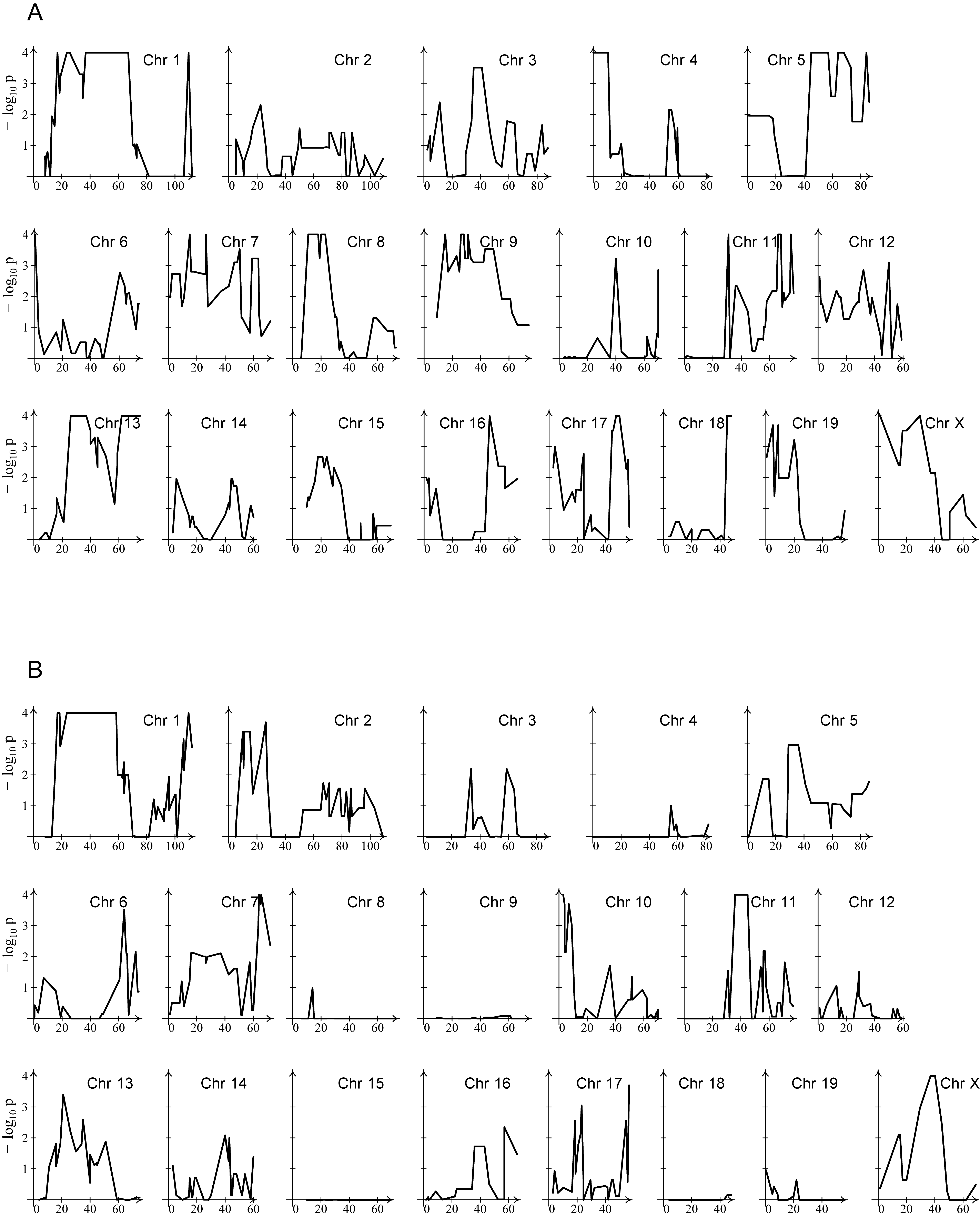

Supplement: Figure S1 — Linkage analysis of early splenic counts independent of the genetic background. Bacillary counts of BCG Russia and BCG Pasteur in the spleen of RC mice at the week 1 time point were used for QTL analysis. AA and BB genotype groups were analyzed without taking into account the gender or genetic background of the RC mice. Significant evidence for linkage was detected across 15 different chromosomes for BCG Russia (A) and across 8 different chromosomes for BCG Pasteur (B) at the week 1 time point. Chromosomal positions are given in centimorgans (cM). (1.83 MB TIF) [file ppat.1001169.s002.tif]

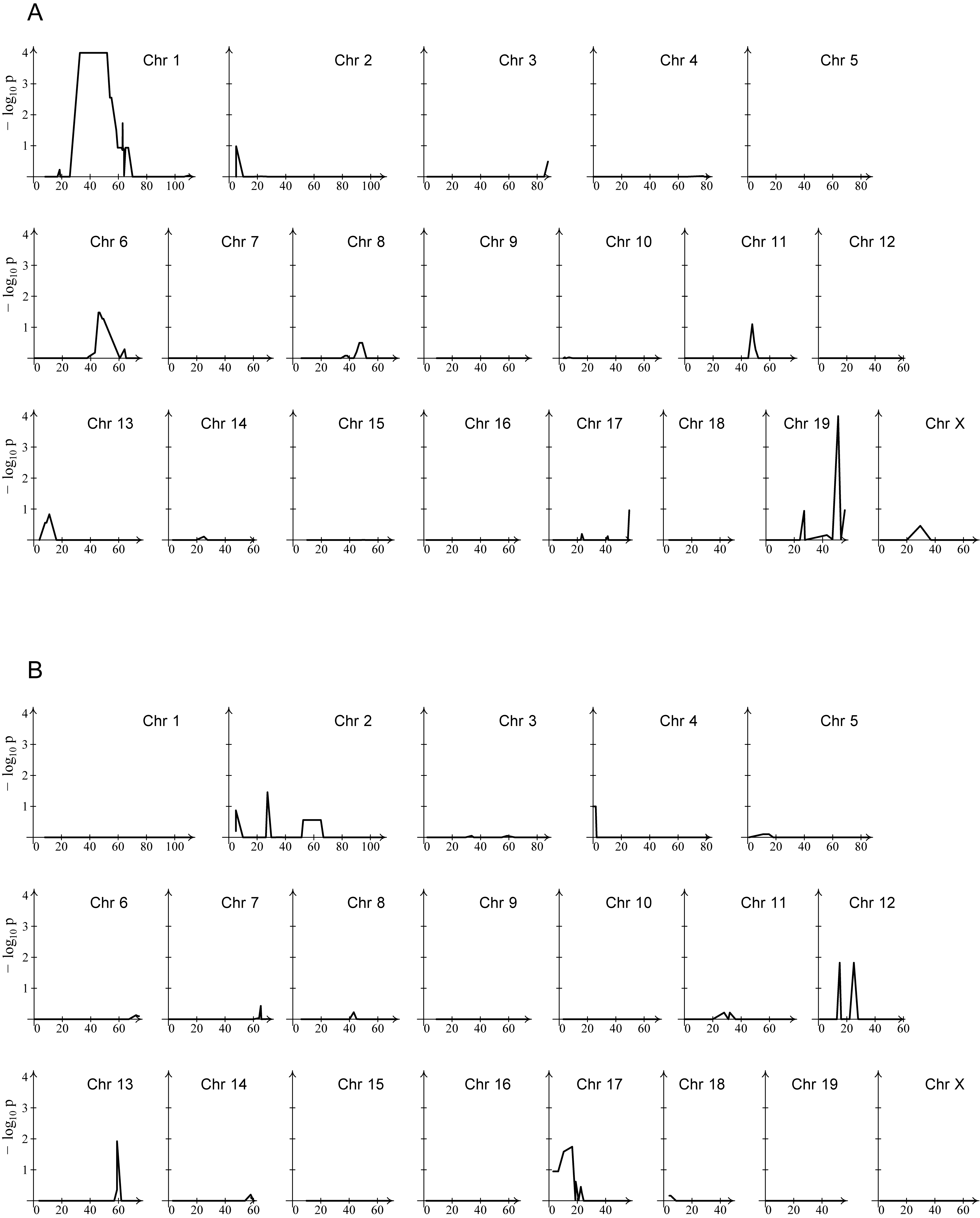

Supplement: Figure S2 — Linkage analysis of late splenic counts independent of the genetic background. Bacterial numbers of BCG Russia and BCG Pasteur in the spleen of RC mice at the week 6 time point were used for linkage analysis. Significant linkages were detected on chromosomes 1 and 19 for BCG Russia (A) whereas no significant evidence for linkage was detected for BCG Pasteur (B). Chromosomal positions are given in centimorgans (cM). (1.59 MB TIF) [file ppat.1001169.s003.tif]

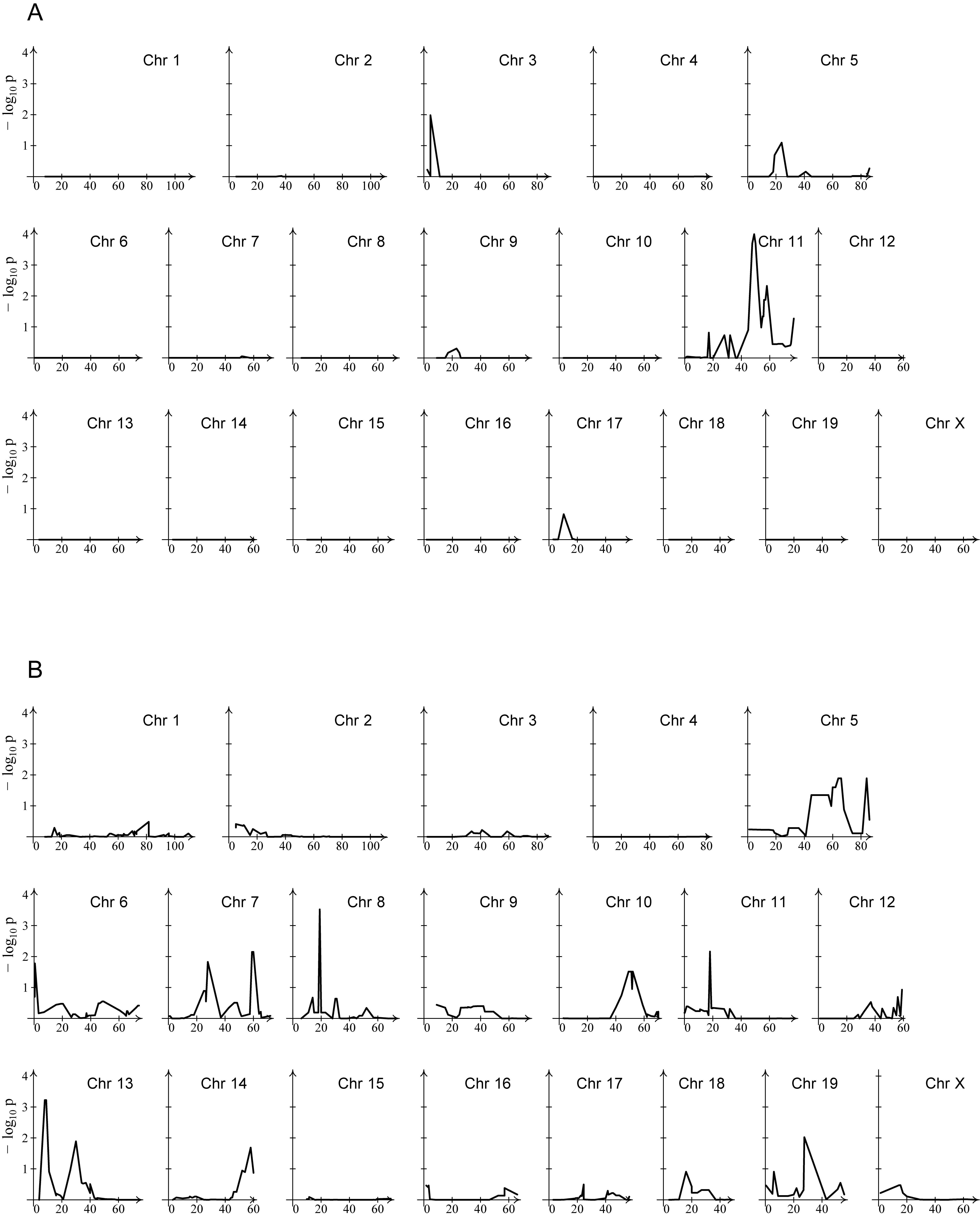

Supplement: Figure S3 — Linkage analysis of late pulmonary counts independent of the genetic background. QTL analysis was performed using pulmonary counts of BCG Russia and BCG Pasteur at the week 6 time point. Loci controlling pulmonary bacterial numbers were identified on chromosome 11 for BCG Russia (A) and chromosome 8 for BCG Pasteur (B). Chromosomal positions are given in centimorgans (cM). (1.61 MB TIF) [file ppat.1001169.s004.tif]

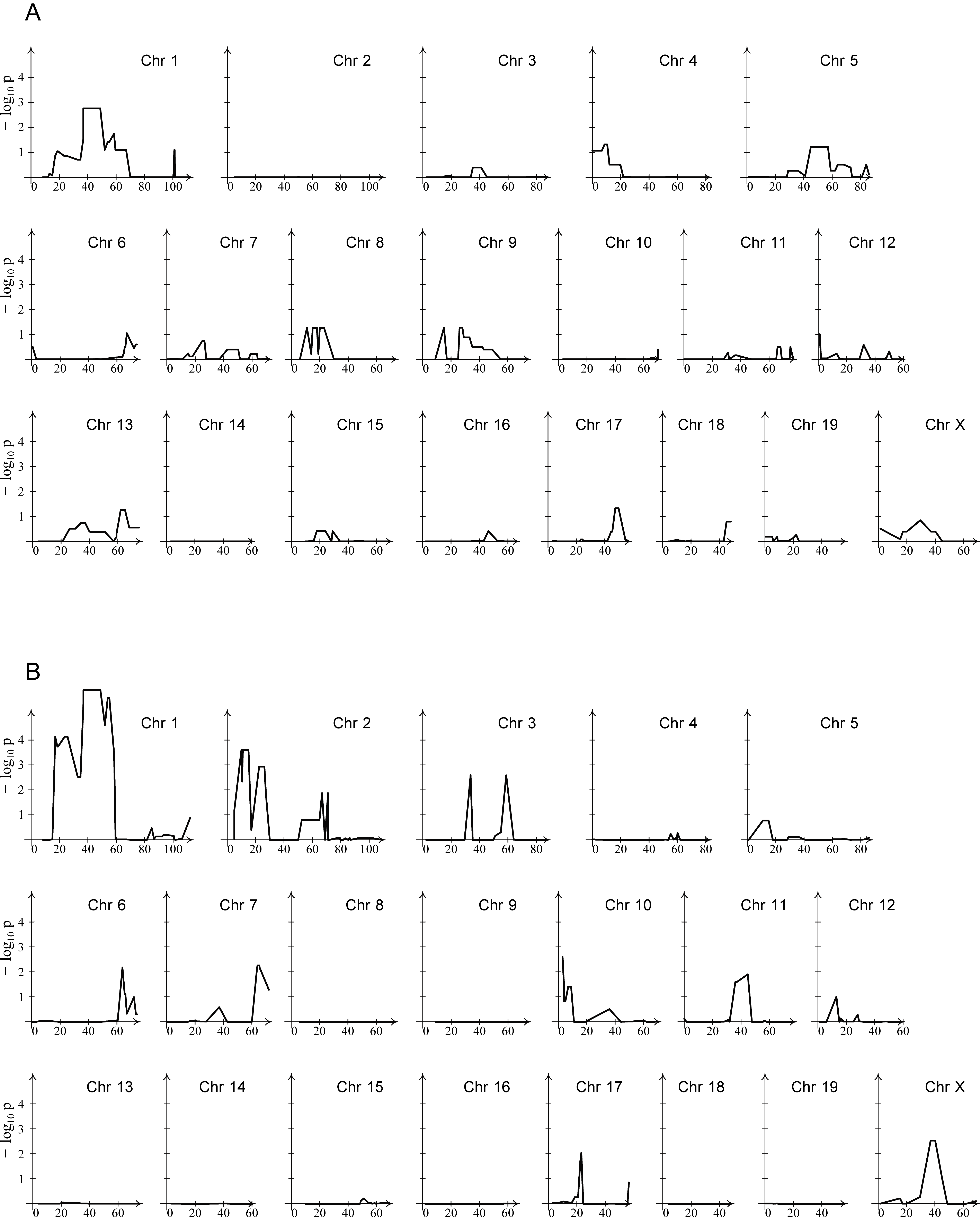

Supplement: Figure S4 — Genetic control of early spleen bacillary counts of BCG Russia and BCG Pasteur. Linkage analysis of splenic bacterial counts at the week 1 time point was performed with an adjustment for strain genetic background and gender. A single locus on chromosome 1 was identified in response to early BCG Russia infection (A). Loci linked to splenic BCG Pasteur counts were detected on chromosomes 1, 2, 3, 6, 7, 10, 17 and X at the week 1 time point (B). Chromosomal positions are given in centimorgans (cM). (1.69 MB TIF) [file ppat.1001169.s005.tif]

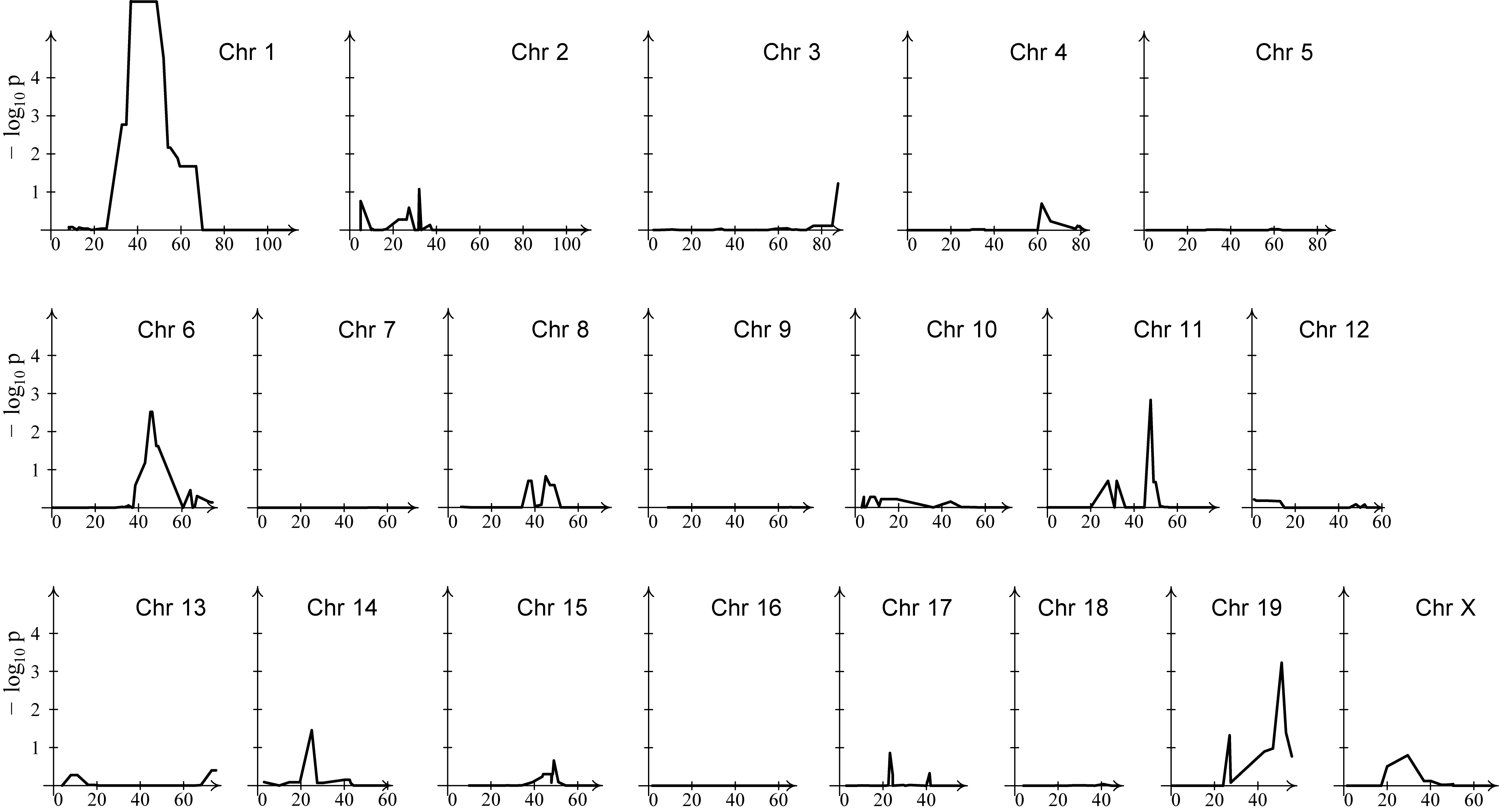

Supplement: Figure S5 — Genetic control of late spleen bacillary counts following infection of the RC strains with BCG Russia. Background- and gender-adjusted QTL analysis was performed using spleen counts of BCG Russia at the 6-week endpoint. A locus on chromosome 1 had a major effect on the bacterial numbers of BCG Russia. Additional loci were detected on chromosomes 6, 11, and 19. Chromosomal positions are given in centimorgans (cM). (0.73 MB TIF) [file ppat.1001169.s006.tif]
